# Male Urethritis in Primary Care: Real-World Evidence From a Portuguese Center

**DOI:** 10.7759/cureus.48028

**Published:** 2023-10-31

**Authors:** Eugénia Pessoa e Costa, Telma Cairrão, Leonor Prata

**Affiliations:** 1 Family Medicine, ACES Amadora, ARS LVT, Amadora, PRT

**Keywords:** quality improvement research, primary care medicine, gonorrhea and chlamydia, sexually transmitted infection (sti), urethritis

## Abstract

Introduction

Early diagnosis and treatment of male urethritis are fundamental for preventing complications and further transmission. Primary healthcare services are the first point of contact for patients. This study aimed to assess the practice and compliance of Unidade Saúde Familiar Conde Lousã (USF CL) with best clinical practices.

Materials and Methods

A retrospective study was undertaken on male urethritis cases at USF CL from January 2018 to March 22, 2021, identified using the International Classification of Primary Care (ICPC) coding system. We established quality criteria encompassing treatment, diagnostics, and reporting. A “sufficiency index” was introduced as a performance metric, which was designed to offer a multiparametric assessment of adherence to these criteria.

Results

Of 77 cases, 60 were included, averaging 27 years of age. In all, 40.7% showed treatment adequacy, with a sufficiency index of 0.28 for epidemiological characterization and 0.52 for co-infection screening. Prevention measures indexed at 0.28, with 27.1% diagnostic notifications.

Conclusion

The study reveals gaps in managing male urethritis at USF CL, underscoring the need for standardized guidelines and better diagnostic tools in primary care. Based on these results, a new multicentric specialized sexually transmitted infections clinic was created within our unit in collaboration with a tertiary hospital [Centro Hospitalar Universitário Lisboa Central (CHULC), Lisbon, Portugal] and the national laboratory center [Instituto Nacional de Saúde Doutor Ricardo Jorge (INSA), Lisbon, Portugal].

## Introduction

Urethritis in men can usually present as a syndrome marked by urethral discharge, dysuria, and erythema of the urethral meatus. It is primarily caused by sexually transmitted infections (STIs) [1] and can be classified as gonococcal, when it is attributed to a *Neisseria gonorrhoeae* infection [2,3], or non-gonococcal, with the majority of cases being attributed to *Chlamydia trachomatis* [3].

Early diagnosis and proper treatment of men with urethritis are of paramount importance to prevent long-term complications of these STIs, such as urethral strictures, epididymitis, and prostatitis [4,5]. Furthermore, with the proper addressing of male patients, there is an associated decrease in female infections, which, although frequently asymptomatic, are also associated with an increased risk of cervicitis, pelvic inflammatory disease, infertility, and ectopic pregnancies [6,7].

While *N. gonorrhoeae* and *C. trachomatis* are the most predominant sexually transmitted bacterial infections in Europe [1,2], no national guidelines delineate a systematic approach to these patients. Consequently, the diagnostic and treatment strategies lean heavily on international recommendations [1-3], which may differ based on the target population they cater to. Nonetheless, they tend to agree that diagnosis typically relies on a direct examination with Gram staining, utilizing a urethral swab, or, in centers devoid of microscopic examination capabilities, nucleic acid amplification test (NAAT) from urine samples with syndromic antibiotic treatment targeting both *N. gonorrhoeae* and *C. trachomatis*.

Family doctors often serve as patients’ primary point of medical contact, making them essential players in the early detection and management of various health conditions, including STIs. However, overcrowded primary care centers where doctors have no specific training in managing STIs or access to microscopic examination or NAAT testing can perform poorly. As such, STI clinics, with properly trained staff and equipment, have proven to be a valid alternative in primary care [8-10].

In Portugal, however, the model of STI clinics remains largely unexplored despite the ongoing limitations in the clinical practice of family doctors. Therefore, this study aimed to provide real-life data on the quality of clinical practice delivered to men with urethritis assessed in the context of a Portuguese primary healthcare unit [Unidade Saúde Familiar Conde Lousã (USF CL)] that serves over 16,000 patients.

Specifically, the study sought the following objectives:

- To assess the adequacy of the treatment being provided.

- To evaluate if these patients’ epidemiological characterization was properly recorded.

- To determine if appropriate diagnostic tests were being ordered in line with each patient’s risk profile.

- To ensure other STIs were being excluded from diagnoses.

- To verify the recording of preventive measures against community transmission of urethritis.

- To measure compliance with the mandatory reporting of urethritis or suspected urethritis cases in the National Epidemiological Surveillance System (SINAVE).

## Materials and methods

This study involved a retrospective evaluation of medical practices related to the management and treatment of male urethritis within the USF CL. Male patients identified under the International Classification of Primary Care (ICPC) coding system with the specific codes U72 (urethritis), Y71 (male gonococcal urethritis), and Y03 (male urethral discharge) were included. This analysis spanned from 2018 to March 22, 2021. Data extraction was undertaken using the Information and Monitoring Module of Functional Units (MIM@UF) facilitated through the SClínico platform and the electronic prescription system (PEM).

To comprehensively assess medical practices, quality criteria based on both the Centers for Disease Control and Prevention (CDC) and the International Union Against Sexually Transmitted Infections (IUSTI) guidelines [1-3] were created (Table 1).

**Table 1:** Appropriate quality criteria set for the approach of men with urethritis HIV, human immunodeficiency virus; HBV, hepatitis B virus; HCV, hepatitis C virus; NAAT, nucleic acid amplification test; STI, sexually transmitted infection; SINAVE, National Epidemiological Surveillance System

Category	Details
(1) Treatment adequacy	- Ceftriaxone 1 g + azithromycin 2 g as a single oral dose or doxycycline 100mg, taken twice daily for 7 days
	- Ceftriaxone 500 mg + azithromycin 2 g as a single oral dose
	- Ceftriaxone 250 mg + azithromycin 1 g as a single oral dose
	- Another therapeutic alternative based on patient comorbidities
(2) Epidemiological characterization of patients	- More than one sexual partner in the past 3 months
	- Irregular use of condoms
	- Men having sex with men
	- Consumption of alcohol or illicit drugs prior to sexual intercourse
	- A past history of another STI
(3) Diagnostic test adequacy	- A request for NAAT urine or urethral exudate or culture of urethral exudate for all patients
	- A request for NAAT/culture of oropharyngeal and anorectal exudate, especially for men having sex with men
(4) Exclusion of other STIs	- Syphilis
	- HIV
	- HBV
	- HCV
(5) Prevention of community transmission of urethritis	- The clinical diary should emphasize the need to inform contacts for testing
	- Advise patients to abstain from sexual activity for a minimum of 14 days post the commencement of treatment
(6) Mandatory reporting compliance in SINAVE	- Documentation of SINAVE notification in the SClínico

**Figure 1:**
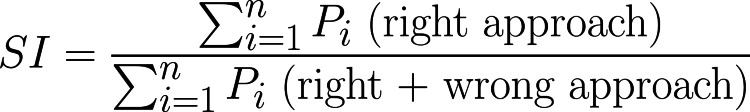
Sufficiency index P*_^i^_* represents the *i*th parameter. *n* represents the total number of parameters you want to consider. The numerator sums up the right approaches for all parameters from P_1_ to P*_n_.* The denominator sums up both right and wrong approaches for all parameters from P_1_ to P*_n_.*

To provide a holistic analysis of performance in quality criteria that encompassed multiple parameters, we used a “sufficiency index” (Figure 1).

This metric served as a composite score, integrating the various determinants of a quality criterion. It was designed to offer a comprehensive insight into the adequacy and effectiveness of healthcare practices. The sufficiency index thus gave a broader view of the overall quality by highlighting the synergy among different components within a specific domain.

## Results

The retrospective assessment of urethritis cases in male patients that occurred between January 1, 2018, and March 31, 2021, at the USF Conde Lousã identified 77 cases that could be included in the study (Table 2). Of these, only 60 cases met the inclusion criteria, with 30 cases of male urethral discharge (ICPC code U72), 15 cases of urethritis (Y71), and 15 cases of gonococcal urethritis (Y03). Of the 17 patients excluded from the analysis, prior medication by another professional was the main reason for exclusion. In these 60 cases included, the patients had an average age of 27 years (16-80 years), with 54 cases evaluated by specialist doctors and only six by internal doctors.

**Table 2:** Summary of male urethritis cases at USF CL from January 1, 2018, to March 31, 2021 *When the existence of more than one consultation record for the same patient and related to the same episode was verified, the clinical record in which the diagnostic hypothesis of urethritis was unequivocally stated was considered for the study, excluding the others. U72, Y71, and Y03 stand for the classification codes registered based on the ICPC coding system. ICPC, International Classification of Primary Care; USF CL, Unidade de Saúde Familiar Conde Lousã

Category	Subcategory	Total
Total cases	-	77
Exclusions	Doctor no longer at USF CL	3
	Medicated externally	6
	SOAP record inconsistency	1
	Duplicate appointment*	4
	Coding error	1
	Diagnostic uncertainty	2
Included cases	Male urethral discharge (U72)	15
	Urethritis (Y71)	15
	Gonococcal urethritis (Y03)	30
Other details	Average age (18-80 years)	27
	Specialist appointments	54
	Resident appointments	6

The previously defined quality criteria were then analyzed.

Regarding the criterion for treatment adequacy, it was observed that appropriate treatment was provided in 40.7% of cases (n = 24). One patient was not empirically treated because a prior diagnostic test was requested. This patient was not counted for the purpose of assessing the treatment adequacy criterion.

For the epidemiological characterization of patients, the analysis considered different parameters. It was observed that in 58.3% of the cases (n = 53), a correct record was taken regarding the patients’ use of condoms; in 48.3% of the patients (n = 29), the record correctly reflected their sexual orientation; in 21.7% of the cases (n = 13), their previous history of STIs was recorded; in 10% of cases (n = 6), inquiries were made about the number of partners in the last three months; and in only 1.7% of consultations (n = 1), there was a record about drug and alcohol consumption. Based on these findings, the overall sufficiency index for this quality criterion was 0.28.

Regarding diagnostic test adequacy, three patients had already undergone this assessment at another institution by the time of their first evaluation at USF CL; therefore, they were not included in this analysis. Of the remaining 57 patients, in 49.1% of cases (n = 28), a urethral exudate culture had been requested. There were no requests for NAAT/urine culture or NAAT/oropharyngeal or anorectal culture. The evaluation of the sufficiency index for this criterion wasn’t conducted since the additional diagnostic tests don’t apply to all patients [the incomplete characterization of our population could introduce a bias in measuring this parameter; for instance, anorectal NAAT is only recommended for the MSM (men who have sex with men) population, and the sexual orientation had been assessed in only 48.3% of the cases].

The quality assessment regarding the screening for other STIs was based on the existence of complementary diagnostic exams (ECD) that ruled out these infections.

From the data, the following observations were made:

- For syphilis infection, records indicated exclusion in 46 cases, which represents 80.7% of the 57 cases assessed. It’s important to highlight that three patients already had results, so they were not counted for the purposes of assessing this quality criterion.

- HIV infection was ruled out in 44 cases, equivalent to 77.2% of the 57 cases.

- HCV infection was excluded in 12 cases, making up 20% of the 57 cases.

- As for HBV infection, exclusion was recorded in 19 cases or 32.8% of the 57 cases. A noteworthy detail is that a record in the clinical diary of a complete HBV vaccination schedule was considered as meeting the quality criteria, thus exempting the request for HBV serology. Furthermore, one patient already had results, and another was a known carrier of chronic HBV disease; hence, these two were not counted for the purposes of assessing this quality criterion.

Taking all factors into account, the overall sufficiency index for this quality criterion was 0.52.

The quality assessment regarding prevention was based on the analysis of information records about the need for testing contacts and the requirement for sexual abstinence for at least 14 days after starting treatment. From the data, it was notable that in 45% of the cases, the patient was informed about the need to test their contacts, but only in 10% of the cases was the importance of avoiding sexual contact documented. The overall sufficiency index was 0.28.

Regarding the need for notification in SINAVE, only 27.1% of cases had this type of notification. One patient was still awaiting ECD results at the time of the consultation, so the decision not to refer was not counted as incorrect and did not contribute to the assessment of this quality criterion.

Table 3 summarizes the previously mentioned multiparameter quality analysis.

**Table 3:** Quality analysis summary *The sufficiency index assessment of this criterion was not performed because the ECDs are not applicable to all patients. STIs, sexually transmitted infections; SINAVE: National Epidemiological Surveillance System

Quality criterion	Score
Appropriate treatment	0.41
Record of epidemiological characterization of patients	0.28
Request for appropriate diagnostic complementary exams	-*
Exclusion of other STIs	0.52
Records on community prevention of urethritis transmission	0.28
Notification in SINAVE of urethritis/suspected urethritis	0.27

## Discussion

The results presented showed a suboptimal approach to male urethritis in USF CL, with relatively low rates of appropriate treatment and recording of epidemiological information. These findings are consistent with the literature [10], confirming the paradox that most STIs are initially diagnosed in primary healthcare settings, but at the same time, professionals in these units often have doubts about their approach.

Indeed, considering all evaluated criteria, excluding other STIs had the best overall sufficiency index (0.52 points), yet it is still far from recommended, considering that excluding other STIs should be carried out in all patients with urethral discharge [1-3]. This fact is further exacerbated by the disparity in screening for these co-infections, particularly the HCV infection, tested only in 20% of cases (compared to 80.7% of patients tested for syphilis).

On the other hand, only 40.7% of patients were correctly medicated. These data seem to align with what has been previously reported for primary care [11, 12], and it has been linked with the therapeutic insecurity felt by family doctors. This insecurity may be even more pronounced in Portugal, where the lack of national guidelines prevents adopting a locally adapted approach for these patients, ultimately increasing therapeutic indecision.

The sufficiency index regarding patients’ epidemiological characterization was only 0.28 points. Although the retrospective nature of the analysis could contribute to this result, the difference in recording between the most recorded parameter (condom use in 58.3% of cases) and the least recorded parameters [drug/alcohol consumption (1.7%), number of recent partners (10%), and previous history of other STIs (21.7%)] might indeed indicate that some of these parameters are overlooked in the epidemiological analysis of these patients. Thus, the importance of establishing criteria for approaching patients suspected of having STIs is emphasized useful not only for symptomatic patients, as those included in the study, but also in screening other patients where the clinical picture may not be as characteristic and where the screening for sexually transmitted pathology is of equal importance.

On the other hand, the incomplete characterization of the patient’s sexual orientation prevented calculating the sufficiency index related to diagnostic test adequacy. Indeed, in only 49.1% of the patients, a microbiological study of urethral exudate was conducted, with no test requested for infection in the oropharynx or anorectum. However, the authors emphasize that even in cases where such identification may have eventually occurred, USF CL doctors had no local resources available to carry out NAAT testing. Given that primary healthcare is indeed one of the main initial STI diagnosis sites [12], it is crucial for doctors to have the appropriate diagnostic means available to halt the community transmission of these infections. However, in Portugal, NAAT tests are only subsidized when performed in a hospital setting. As reinforced by the data from this study, this has hindered a proper approach to these patients. As such, the authors postulate that decentralizing subsidized testing to a community network of STI clinics or dedicated primary care centers could mitigate the lack of proper STI testing and thus improve the overall care of these patients.

Regarding prevention, it’s noteworthy that the need for contact testing was only mentioned in 45% of cases, and only in 10% was sexual abstinence recommended, although the retrospective nature of the analysis could influence the correct assessment of this criterion.

Finally, the notification in SINAVE (platform for mandatory infectious disease notification) was not mentioned in 72.9% of cases. The sufficiency index for this criterion was only 0.27 points, which might be explained by the fact that notification is mandatory at diagnosis, but there’s no certainty of that diagnosis from a microbiological standpoint at the time of patient contact. This fact is compounded by the lack of available NAAT tests for the doctor and that patients might not carry out the ECDs, hindering diagnostic confirmation and significantly contributing to underreporting, which would be mandatory.

Regarding the study’s limitations, its retrospective nature is evident and may have resulted in an incomplete classification of some consultation episodes, contributing to the limited number of included patients. However, this can also be attributed to the COVID-19 pandemic, which influenced the study’s time frame and subsequently restricted the access of non-COVID patients to primary healthcare services. Despite these limitations, to the best of the authors’ knowledge, this study remains valuable as it’s the first in Portugal to probe into the quality of care for this group of patients, underlining the necessity to enhance the approach toward Portuguese men with urethritis.

Based on the study’s results, the authors proposed the creation of a specialized consultation for STIs within the scope of primary care. This consultation was established in collaboration with the specialized services of a tertiary referral hospital, specifically the Dermatovenereology Department of Santo Antonio dos Capuchos Hospital, Lisbon, Portugal, and in partnership with the National Institute of Health Doctor Ricardo Jorge (INSA), Lisbon, Portugal. This framework ensured comprehensive training and provided the capability to refer more severe cases while granting access to essential diagnostic resources. The literature has consistently underscored the significance of such integrated care, emphasizing the benefits of specialized consultations that are decentralized from major hubs and positioned closer to patients [13-15]. Recognizing this, the authors established a patient-centric approach, seen as vital for effectively managing patients with STIs. With this strategy in place, they hope to improve healthcare delivery to their patients significantly.

## Conclusions

This study showed that there was a pressing need for overall improvement in the clinical approach to male urethritis by the physicians at USF CL. The absence of national clinical guidance and the challenges in accessing the necessary diagnostic tests might have explained some of the observed findings. Based on these results, a new multicentric specialized STI clinic was created within USF Conde Lousã in collaboration with a tertiary hospital and the national laboratory center. This new approach aims to enhance the training of primary care physicians and allow the provision of NAAT tests within this community, thus improving the overall care for STI patients.
